# ParaDockS – an open source framework for molecular docking

**DOI:** 10.1186/1758-2946-4-S1-F3

**Published:** 2012-05-01

**Authors:** Martin Pippel, Michael Scharfe, Renè Meier, Wolfgang Sippl

**Affiliations:** 1 Institute of Pharmacy, Martin-Luther-Universität Halle-Wittenberg, Halle (Saale), 06120, Germany; 2 Institute of Biochemistry, University of Leipzig, Leipzig, 04103, Germany

## 

The generation and evaluation of molecular complexes in order to predict binding modes of protein ligand complexes, is still a challenging task. Many different algorithms and approaches try to solve the molecular docking problem. Within this work we introduce the open source docking platform ParaDockS (Parallel Docking Suite) [1], that allows the convenient incorporation of existing and new approaches either to describe ligand-receptor interaction or to search for native poses.

The goal of this work pursues the line of thought, that ParaDockS is open source. We want to initiate a highly dynamic community, so that both users and developers contribute with their knowledge and experience to take this very interesting field one step further.

The framework, written in C++, is highly modular designed. This offers an easy access to developers to work only on their area of expertise. ParaDockS provides a functionality to analyze, process and store different kind of input structures.

Another focus is set on the implementation of scoring functions. Beside implemented scoring functions PScore, PMF04 and Drugscore, ParaDockS offers the possibility to derive and apply target specific scoring functions. Therefore, we implemented a workflow to derive target class specific PMF atom-pair potentials. This involves the preparation of complex structures to get an adequate structural database. The definition of atom types and the derivation of pair potentials can completely be accomplished in the upcoming version of ParaDockS. The resulting atom pair potentials can be directly used as scoring function for docking or rescoring approaches. The whole workflow was applied and validated for kinases, but is applicable to every target class with enough structural data.

One of the latest improvements involves an interface to the pharmacophor modeling program LigandScout [3]. This offers the big advantage to setup the docking with a great graphical interface, as well the created pharmacophores can be used to guide the docking process.
